# Compensation for Avian Influenza Cleanup

**DOI:** 10.3201/eid1302.061391

**Published:** 2007-02

**Authors:** Sayako Kanamori, Masamine Jimba

**Affiliations:** * University of Tokyo, Tokyo, Japan

**Keywords:** avian influenza, compensation, developing country, global health, health policy, letter

**To the Editor:** Since 2003, highly pathogenic avian influenza (HPAI) H5N1 has shaken the world. In 10 countries, 258 confirmed cases in humans and 154 deaths have been reported (*1*). The number of countries with confirmed HPAI in poultry and wild birds jumps to 54 (*2*). Almost all persons infected with H5N1 have had close contact with sick or dead poultry by having butchered them, plucked them, or played (children) with them (*3*). Because H5N1 can potentially mutate or reassort into a strain capable of efficient human-to-human transmission, rapid elimination of the H5N1 virus in poultry and other risk-reduction interventions are thought to be essential for preventing further spread of HPAI (*4*). As a result, thousands of workers around the world have culled millions of domestic poultry (*5*).

Preemptive culling creates a major concern with regard to compensation. In Nigeria, for example, affected farmers have yet to be compensated >50 million Nigerian Naira (>US$ 0.4 million) because of the ministry’s cash flow problems (*6*). On the other hand, US poultry farmers who participate in a US Department of Agriculture (USDA) program to prevent the spread of disease would be fully compensated for loss of poultry and equipment if even a low-pathogenic strain of avian influenza were found in the United States (*7*). This rule not only strengthens US protection against avian influenza but also minimizes any negative effect on the US poultry trade.

As discussed by the World Bank (*8*), the situations of these 2 countries raise several questions: Who should pay the compensation? For what should compensation be paid? Who should be compensated? With regard to the first question, each country’s government is an exclusive funding source. However, in Nigeria, the amount of compensation overwhelms the government’s capacity. Some countries, like Australia, may get additional funding from alternative sources such as private sectors, regional economic groups, or international funds (*9*). Because national resources are often scarce, most developing countries must rely on international donors for a great deal of the funding for compensation programs.

The response to the second question, extent of compensation, varies. In Nigeria, farmers are partially compensated for loss of poultry; however, in the United States, farmers who are part of the USDA program are fully compensated for loss of poultry and equipment. Setting the amount of compensation is difficult and can affect the outcome of culling efforts. In Thailand, to take advantage of the program in which compensation was perceived as high, some farmers reportedly moved infected poultry into previously uninfected areas. In Vietnam, where compensation was perceived as low, culling compliance was poor (*8*).

The last question, who should be compensated, seems straightforward for the United States, where only farmers who participate in the USDA program would be fully compensated. However, H5N1 does not affect only farmers who sign up for such a program. And not all poultry are raised in commercial operations, especially in developing countries. In Thailand, for example, >80% of infected poultry are reportedly raised in backyards (*10*). Reasonable assumptions are that those backyard farmers do not honestly report dying poultry or that they rush sick and dying poultry to market, causing the disease to spread. Additional questions revolve around potential compensation for those who are involved in the poultry industry but who do not own poultry (e.g., poultry processing plant operators and their staff).

Because each country’s needs and circumstances differ, building a coherent plan for tackling HPAI is difficult. However, each stakeholder should consider compensation as part of an overall package of prevention, preparedness, and response strategies toward controlling and preventing the spread of HPAI. Because H5N1 does not respect international boundaries, donors worldwide should step forward to support the most affected and vulnerable developing countries.

## References

[1] World Health Organization. Affected areas with confirmed cases of H5N1 avian influenza since 2003: status as of 29 November 2006 [cited 2006 Dec 16]. Available from http://gamapserver.who.int/mapLibrary/Files/Maps/Global_H5N1inHumanCUMULATIVE_FIMS_20061129.png

[2] World Organization for Animal Health. Update on avian influenza in animals (type H5): 14 December 2006 [cited 2006 16 Dec]. Available from http://www.oie.int/downld/AVIAN%20INFLUENZA/A_AI-Asia.htm

[3] World Health Organization. Avian influenza (“bird flu”)—fact sheet: February 2006 [cited 2006 Oct 25]. Available from http://www.who.int/mediacentre/factsheets/avian_influenza/en/#history

[4] Food and Agriculture Organization, World Organization for Animal Health. Global strategy for the progressive control of highly pathogenic avian influenza (HPAI), November 2005. [cited 2006 16 Dec]. Available from http://www.fao.org/docs/eims/upload//210745/Glo_pro_HPAI_oct05_en.pdf

[5] Lahariya C, Sharma AK, Pradhan SK. Avian flu and possible human pandemic. Indian Pediatr. 2006;43:317–25. 16651670

[6] Bird flu: govt suspends compensation to farmers. The Tide Online, Saturday, Oct 7, 2006 [cited 2006 Oct 25]. Available from http://www.thetidenews.com/article.aspx?qrDate=10/07/2006&qrTitle=Bird%20flu:%20Govt%20suspends%20compensation%20to%20farmers&qrColumn=NEWS

[7] USDA expands compensation for bird flu cleanup. Reuters, Fri, Sep 22, 2006 [cited 2006 Oct 25]. Available from http://today.reuters.com/news/articlenews.aspx?type=domesticNews&storyID=2006-09-22T221333Z_01_N22219184_RTRUKOC_0_US-BIRDFLU-USDA-CLEANUP.xml&archived=False

[8] The World Bank. Enhancing control of highly pathogenic avian influenza in developing countries through compensation: issues and good practices [cited 2006 Dec 13]. Washington (DC): World Bank; 2006. Available from http://siteresources.worldbank.org/INTARD/Resources/HPAI_Compensation_Final.pdf.

[9] Beers P. Compensation arrangements in Australia [cited 2006 Oct 25]. Presented at Capacity Building Seminar on Avian Influenza: Preventing AI at Its Source and a Dialogue on Indemnity; 2006 Sep 12–13; Hoi An, Viet Nam. Available from http://www.apec.org/apec/documents_reports/health_task_force/2006.MedialibDownload.v1.html?url=/etc/medialib/apec_media_library/downloads/taskforce/HTF/mtg/2006/pdf.Par.0075.File.v1.1

[10] Tiensin T, Chaitaweesub P, Songserm T, Chaisingh A, Hoonsuwan W, Buranathai C, Highly pathogenic avian influenza H5N1, Thailand, 2004. Emerg Infect Dis. 2005;11:1664–72. 16318716 10.3201/eid1111.050608 PMC3367332

